# Rebleeding after Stent Grafting for the Celiac Artery Bleeding following Extended Pancreaticoduodenectomy

**DOI:** 10.1155/2013/781698

**Published:** 2013-12-08

**Authors:** Kengo Ohta, Masashi Shimohira, Takuya Hashizume, Tatsuya Kawai, Masahiro Muto, Junichi Honda, Yuta Shibamoto

**Affiliations:** Department of Radiology, Graduate School of Medical Sciences, Nagoya City University, Nagoya 467-8601, Japan

## Abstract

We report a 74-year-old man with rebleeding following stent grafting for the celiac artery bleeding which developed after extended pancreaticoduodenectomy for cancers of the bile duct and stomach. The site of rebleeding seemed to be different from the site of the first bleeding, so it was considered not an endoleak but a new bleeding. It was successfully treated by placement of another stent graft.

## 1. Introduction

Bleeding after pancreaticoduodenectomy is a fatal complication, and endovascular treatment is widely performed. Recently it has been reported that stent grafting is especially an appropriate treatment [1]. Here, we present a rare complication of rebleeding developing 40 days after stent graft placement in the celiac artery.

## 2. Case Report

**Figure 1:**
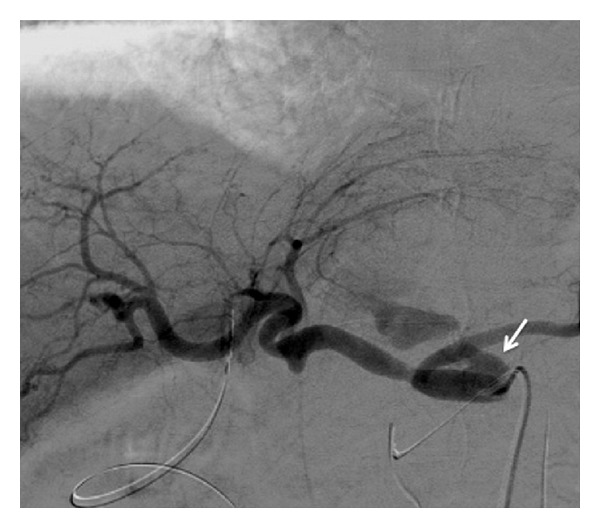
Angiography shows bleeding from celiac artery (arrow).

**Figure 2:**
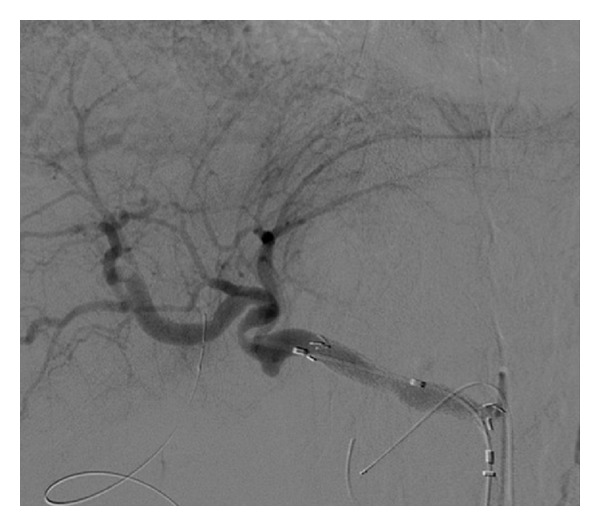
Angiography shows successful placement of the stent graft.

**Figure 3:**
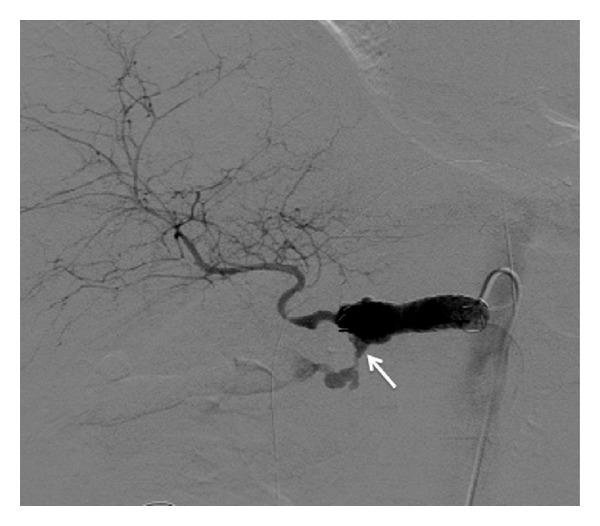
Angiography shows rebleeding from a distal site apart from the first bleeding site (arrow).

**Figure 4:**
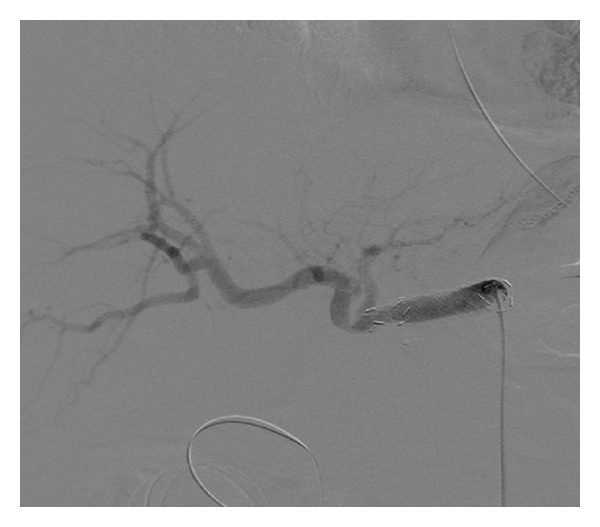
Angiography shows successful placement of the stent graft at the inside of the initial stent graft to the distal site.

A 74-year-old man with distal bile duct cancer and gastric cancer underwent extended pancreaticoduodenectomy. Thereafter, leakage of pancreatic juice occurred, for which drainage treatment was performed. At 12 days after surgery, a bleeding from the drainage tube and low blood pressure were observed, so an emergency angiography was performed. A bleeding from the celiac artery was observed on angiography (Figure 1), and a 5 Fr balloon catheter was inflated in the celiac artery for temporary hemostasis, because we needed some time to discuss with surgeons and to prepare a stent graft. Then, a stent graft (Niti-S; Century Medical, Tokyo, Japan) with a diameter of 10 mm and a length of 5 cm was placed at the celiac artery to the common hepatic artery successfully (Figure 2). Resection of the residual stomach, residual pancreas, and spleen was immediately performed following the stent grafting. After that, wound infection and insufficient anastomosis of the esophagus and jejunum were observed; leukocyte count was 5,400/*μ*L and C-reactive protein level was 5.4 mg/mL. So, drainage treatment was performed. At 40 days after this stent grafting and surgery, melena and reduction of blood pressure were observed. Therefore, emergency angiography was performed, which revealed a bleeding from the celiac artery (Figure 3). It seemed to be from a distal site apart from the site of the first bleeding, and it was considered not a leakage but a new bleeding. Under fluoroscopy, it was confirmed that the stent graft was not broken. Temporary balloon hemostasis was performed again. Then, we discussed with our surgeon and decided to perform the stent grafting, because it seemed really difficult to reach the celiac artery under laparotomy due to strong adhesion caused by the previous operation. Thereafter, the stent graft (Niti-S) with a diameter of 10 mm and a length of 6 cm was placed at the inside of the initial stent graft to the distal site successfully (Figure 4). There was no recurrence of bleeding during a 3-month followup period. No antithrombotic therapy was given during follow-up.

## 3. Discussion

Delayed arterial bleeding after pancreaticoduodenectomy is not common, but a potentially fatal complication [2–4]. Massive arterial bleeding occurs as a result of inflammatory vascular erosion related to pancreatic juice or bile leaking from an insufficient anastomosis or local infection. Surgical exploration and identification may be difficult in acute situations and hazardous because of adhesions and surrounding postsurgical tissue friability [5, 6]. Thus, the endovascular treatment such as transcatheter arterial embolization (TAE) or stent grafting is widely performed. Gwon et al. [1] compared TAE and stent grafting for extrahepatic artery bleeding after pancreatobiliary surgery. They reported that two patients in the TAE group died of hepatic failure and multiorgan failure within 7 days. Furthermore, hepatic ischemia and infarction were observed in six (33%, 6/18) and eight (44%, 8/18) patients, respectively, and hepatic abscess with hepatic infarction was observed in one patient (5.6%, 1/18). On the other hand, early stent thrombosis with bile duct necrosis was observed in one patient in the stent grafting group (14%, 1/7). They concluded that stent grafting is better than TAE for preserving intrahepatic arterial flow.

In this case, therefore, we did not perform TAE but performed stent graft placement after the first angiography. At second angiography, however, it was not easy to decide treatment strategy, because the bleeding seemed to have occurred at a distal site apart from the first bleeding site. So, we thought it was caused by abdominal infection following intestinal insufficient anastomosis. Thus, at that time, we considered TAE instead of stent grafting because the common hepatic artery might have been involved by inflammation and fragility. However, as a result of discussion with the surgeon, we decided to perform stent grafting to protect the liver, and if rebleeding occurs again, we will perform TAE.

A few complications after stent graft treatment were reported [1–3]. Rebleedings due to the dislodgment of stent grafts have been reported but most of them occurred within a day [2]. We experienced delayed bleeding at 40 days after stent graft treatment. Thus, one should be aware of this complication, and careful observation after stent grafting with infection is recommended.
